# A standalone bioreactor system to deliver compressive load under perfusion flow to hBMSC-seeded 3D chitosan-graphene templates

**DOI:** 10.1038/s41598-019-53319-7

**Published:** 2019-11-14

**Authors:** Joseph Lovecchio, Paolo Gargiulo, Jose Luis Vargas Luna, Emanuele Giordano, Ólafur Eysteinn Sigurjónsson

**Affiliations:** 1 Institute of Biomedical and Neural Engineering, Reykjavík University, Menntavegur 1, 101 Reykiavík, Iceland; 2 Laboratory of Cellular and Molecular Engineering “Silvio Cavalcanti” - Department of Electrical, Electronic and Information Engineering “Guglielmo Marconi” (DEI), University of Bologna, Via Cesare Pavese 50, 47522 Cesena, FC Italy; 3 Advanced Research Center on Electronic Systems (ARCES), University of Bologna, Via Vincenzo Toffano 2/2, 40125 Bologna, Italy; 4 Department of Science, Reykjavík University, Menntavegur 1, 101 Reykiavík, Iceland; 5 Health Sciences and Technologies - Interdepartmental Center for Industrial Research (HST-ICIR), University of Bologna, Via Tolara di Sopra 41/E, 40064 Ozzano dell’Emilia, BO Italy; 6 The Blood Bank, The Landspitali University Hospital, Snorrabraut 60, 105 Reykjavík, Iceland; 7 Present Address: Center of Medical Physics and Biomedical Engineering, Medical University of Vienna, Waehringer Guertel 18-20/4L, 1090 Wien, Austria

**Keywords:** Tissue engineering, Biomedical engineering

## Abstract

The availability of engineered biological tissues holds great potential for both clinical applications and basic research in a life science laboratory. A prototype standalone perfusion/compression bioreactor system was proposed to address the osteogenic commitment of stem cells seeded onboard of 3D chitosan-graphene (CHT/G) templates. Testing involved the coordinated administration of a 1 mL/min medium flow rate together with dynamic compression (1% strain at 1 Hz; applied twice daily for 30 min) for one week. When compared to traditional static culture conditions, the application of perfusion and compression stimuli to human bone marrow stem cells using the 3D CHT/G template scaffold induced a sizable effect. After using the dynamic culture protocol, there was evidence of a larger number of viable cells within the inner core of the scaffold and of enhanced extracellular matrix mineralization. These observations show that our novel device would be suitable for addressing and investigating the osteogenic phenotype commitment of stem cells, for both potential clinical applications and basic research.

## Introduction

Tissue engineering (TE) strategies aiming at enhancing bone repair and regeneration are anticipated to become a standard clinical treatment in the future^1^. However, consensus has yet to be reached about fast, clean, and reproducible procedures to precondition tissue engineered (TEed) constructs towards the bone phenotype. Bone native tissue is regularly subjected to mechanical cues, such as bending and compression elicited by physical activity, and to shear stress resulting from interstitial fluid movement as a consequence of loading^2^. The conversion of these physical stimuli into molecular signals and biological responses, which is referred to as mechanotransduction, drives molecular cascades of events triggering bone remodelling^3,4^. Accordingly, an appropriate mechanical loading of cells in culture promotes their expansion^5^, along with mineralized extracellular matrix (ECM) production^6–8^. Cell physical triggering can be obtained via dedicated actuators - usually referred to as bioreactor systems - addressing a specific tissue phenotype according to their setup configuration^9–17^. Thus, including the processing of a bone TEed construct in a dedicated bioreactor system might be a viable translational medicine strategy. Building on our experience^8,18–22,41^ we have developed a standalone perfusion and compression bioreactor system to enhance the cell viability and ECM mineralization of mesenchymal stem cells (MSCs) cultured within 3D chitosan graphene (CHT/G) scaffold templates. Other devices have been described in the literature^23–27^, but none of these can simultaneously provide standalone incubator functionality, automatic media exchange, and online monitoring of the TEed construct.

The bioreactor system described herein uses 3D-printed custom chambers to host the tissue constructs. These allow the administration of perfusion flow through a dedicated peristaltic pump and application of a compressive axial deformation through a custom-made mechanical loading platform driven by a precise stepper motor. Moreover, an automatic media replacement system is included to minimize the cell culture contamination risk. Finite element analysis was used to determine the perfusion and compression input parameters to be administered to the TEed constructs. Enhanced cell viability and initial ECM mineralization was evident as early as 1 week into treatment, indicating that this prototype may be suitable to implement as a standard method for rapidly inducing and/or investigating osteogenic phenotype commitment.

## Results

The bioreactor system detailed in this manuscript is a novel standalone device that allows culturing of 3D cell constructs within a controlled environment, where tightly regulated medium perfusion and scaffold compression are intended to sustain human bone marrow-derived mesenchymal stem cell (hBMSC) survival, proliferation, and commitment towards an osteogenic phenotype.

**Figure 1:**
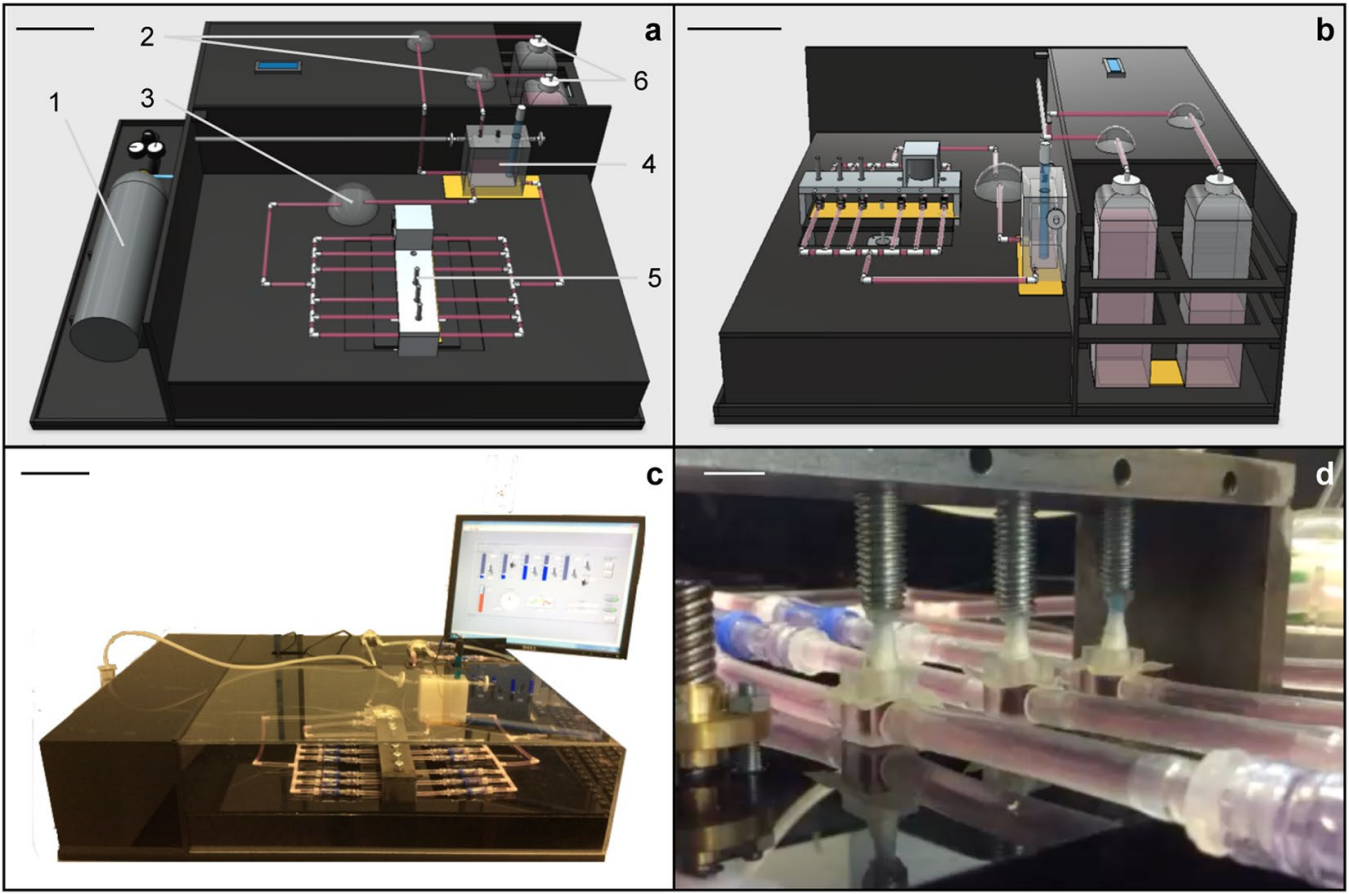
Bioreactor system layout. (**a**) Equipment diagram showing the front view of the bioreactor system set-up. 1: CO_2_ tank; 2: automatic media replacement peristaltic pumps; 3: perfusion peristaltic pump; 4: measurement chamber with temperature and pH sensors inside and heating pad below; 5: mechanical loading unit; 6: fresh and waste media bottles. (**b**) Side view of the bioreactor system (see (**a**) for details); (**c**) Actual bioreactor prototype; (**d**) Detail of the cell culture perfusion/compression unit. Scale bars: (**a**–**c**) = 10 cm; (**d**) = 1 cm.

**Figure 2:**
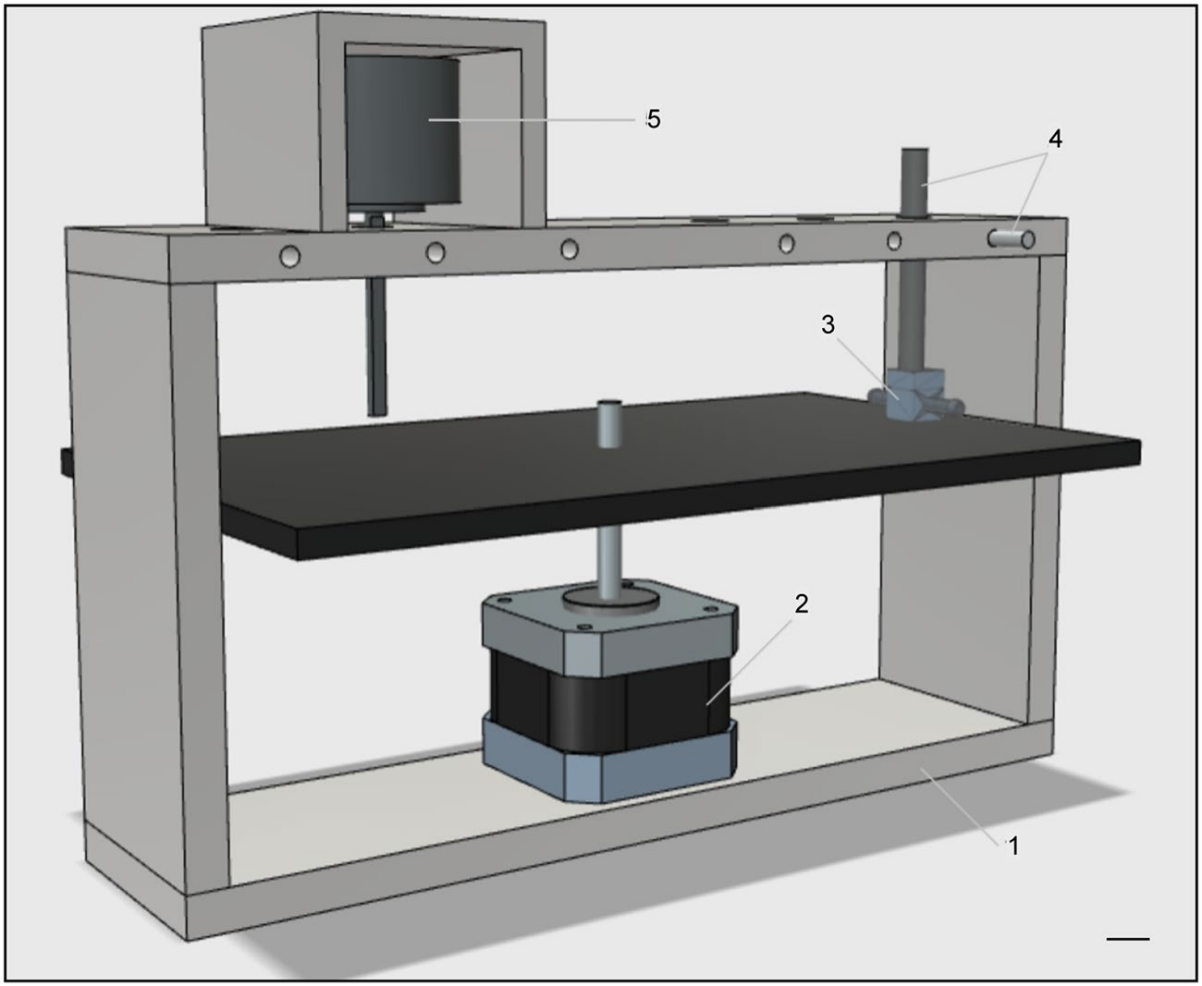
Mechanical loading unit. Layout of the unit used to apply mechanical compression onto seeded scaffolds hosted within the custom-made chambers. 1: aluminium case (20 × 5 × 12 cm); 2: stepper motor driving the attached compression plate; 3: custom-made chamber; 4: piston and piston locker; 5: load cell. Scale bar: 1 cm.

**Figure 3:**
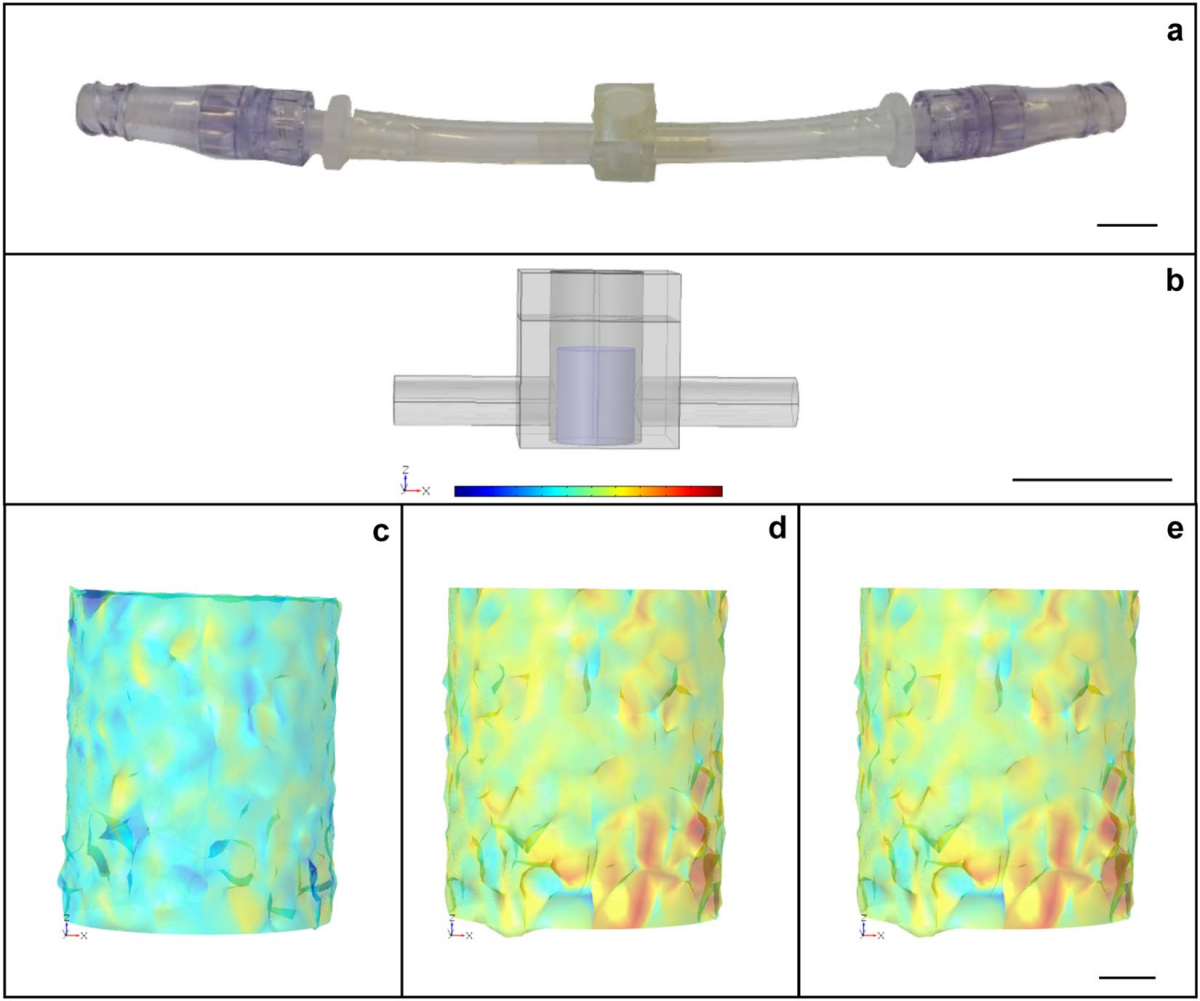
Perfusion/compression culture chamber hosting a 3D scaffold. (**a**) Removable culture unit. (**b**) Finite element model (FEM)-modelled chamber (grey) and scaffold (blue). Also shown are the stresses impacting over the scaffold when (**c**) perfusion (1 mL/min), (**d**) compression (1% axial deformation), and (**e**) perfusion and compression (1 mL/min and 1% axial deformation) are applied. In (**c**), the predominance of a light blue color shows low-shear stress with average values of about 10^−6^ Pa. In (**d**,**e**), the predominance of a yellow/orange color distribution shows higher-compressive stresses, with average values of about 100 Pa.

**Figure 4:**
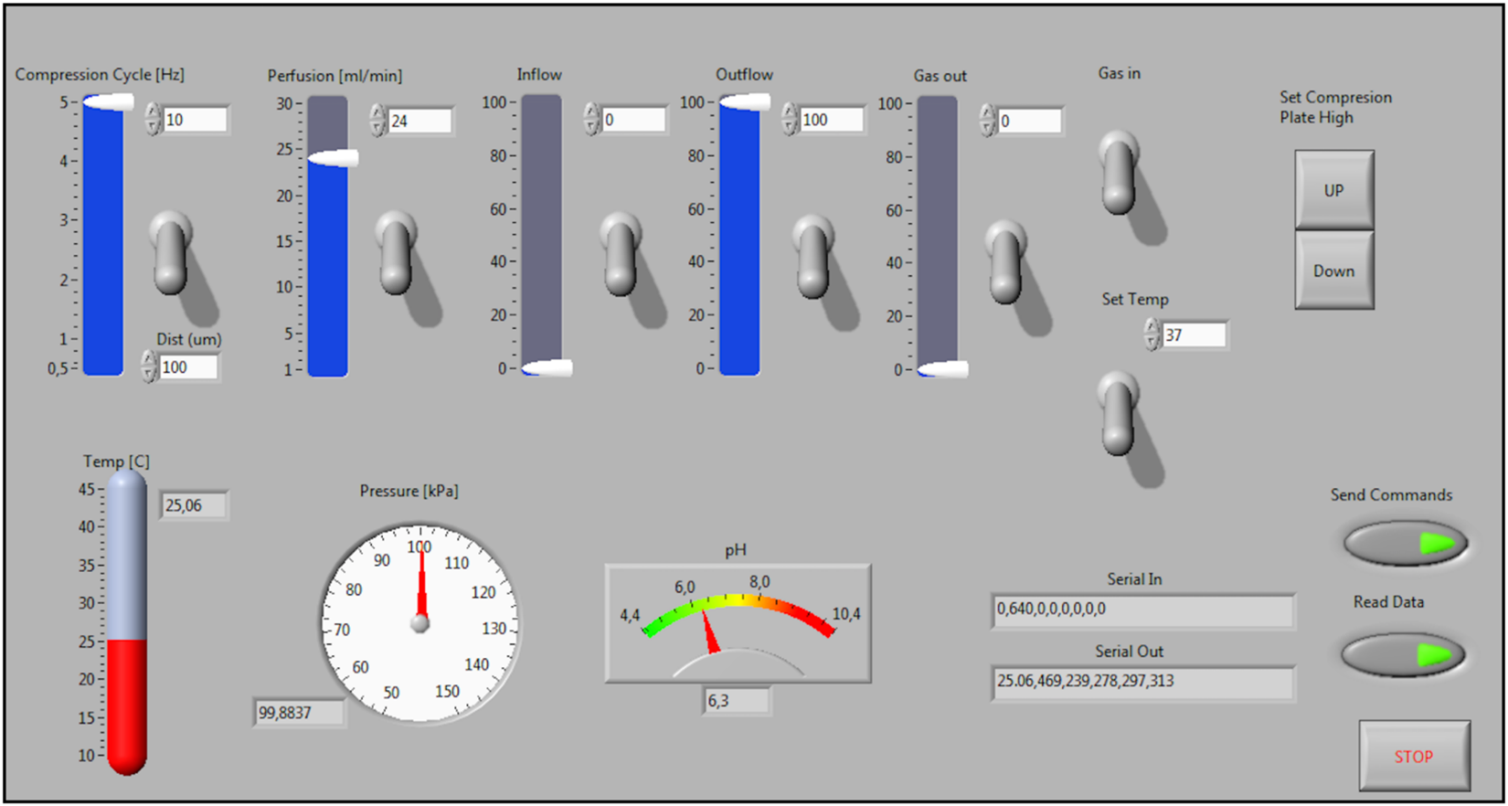
Graphical User Interface. On/off switches, sliders and type-in boxes easily tune all the actuators; up/down buttons align the scaffolds to the pistons; temperature, pressure and pH indicator show all the operative parameters deriving from the sensors; send, read and stop buttons set the communication to/from Arduino.

**Figure 5:**
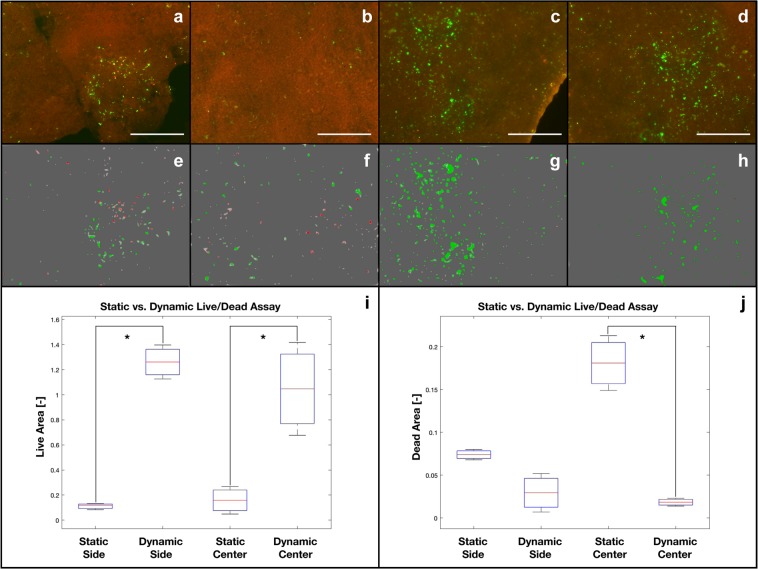
Cell viability/proliferation within the 3D CHT/G scaffold under either static (control; **a** = (outer layer; **b** = inner layer) or dynamic (perfusion and compression; **c** = outer layer; **d** = inner layer) culture conditions. Corresponding maximum entropy threshold-based image segmentation is shown (**e**,**f** = static; **g**,**h** = dynamic). Quantitative analysis of the total amount of scaffold area covered by the live cells (green areas) and dead cells (red areas) is also shown (**i** = live cells; **j** = dead cells, where “side” corresponds to the outer layer and “center” corresponds to the inner layer). *Statistically significant difference (Student’s t-test; p < 0.05). Scale bar 50 μm.

In its final layout, the prototype bioreactor system, shown in Fig. 1c, consists of a sturdy unibody Plexiglas case (with dimensions of 80 × 60 × 25 cm, length × width × height) containing all the components for operating the relevant mechanical actuation. Specifically, perfusion through the biocompatible materials used for the cell interfacing environment (silicon Tygon^®^ tubing and polymeric TuskT^®^ culture units) is driven by a WELCO WP10 peristaltic pump (Fig. 1a). Compression is actuated via a custom-made aluminium mechanical loading unit (with dimensions of 20 × 5 × 12 cm; Figs 1a–5,d and 2) equipped with a Songyang NEMA-17 hybrid bipolar stepping motor delivering frequency- and displacement-controlled axial deformation.

To this aim, the motor includes a lead screw (Fig. 2–2) that drives the vertical movement of an integrated plate hosting multiple culture units/chambers on top (Fig. 2–3). These are in contact with adjustable pistons (Fig. 2–4) intended to compress the scaffolds contained within the chambers along with the plate strokes. The design includes the option of a load cell (Fig. 2–5) that can be used for online monitoring of the scaffold Young’s modulus.

**Figure 6:**
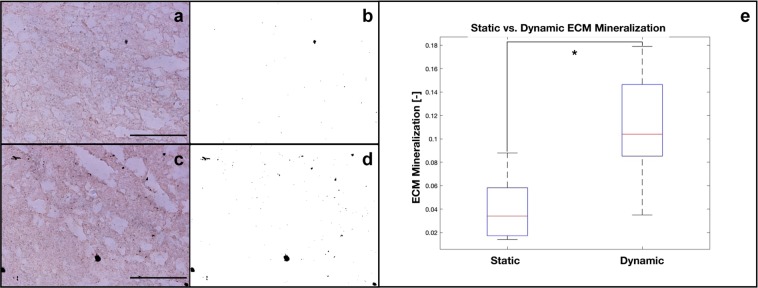
Optical microscopy images of ECM mineralization (Von Kossa staining) within the core of the 3D CHT/G scaffold under either static (**a**) or dynamic (perfusion and compression) (**c**). Corresponding maximum entropy threshold-based image segmentation is also shown (**b**,**d**). Quantitative analysis of the total amount of scaffold area covered by the von Kossa black spots is shown (**e**). *Statistically significant difference (Student’s t-test; p < 0.05). Scale bar = 250 μm.

Up to six 300-µL culture units (Fig. 3a) can operate in parallel alignment (Fig. 1a–5). Each culture chamber can host a single 3D scaffold, maintained under continuous medium flow and cyclic compression. Each culture unit can easily be disconnected from the perfusion line, thanks to the use of Spiros^®^/MicroClave^®^ connectors that allow the risk of leakage from the fluid circuit (Fig. 3a) to be avoided. This last feature supports the option of carrying out additional vital analysis of the TEed constructs outside of the apparatus; the culture units can later be reconnected to the apparatus to resume mechanical stimulation. The medium temperature and pH are constantly monitored and maintained at 37 °C and 7.4, respectively, within the device. This removes the need for a dedicated cell culture incubator, allowing the device to be located on a standard lab benchtop. Culture medium replacement is automatically carried out by additional peristaltic pumps (Fig. 1a–2), which remove 80% of the exhausted medium, by volume, into the waste media bottle and add the same quantity of fresh medium from the fresh media bottle (Fig. 1a–6,b) every three days.

A user-friendly graphical user interface (GUI) (Fig. 4) allows easy tuning of all relevant parameters (i.e. temperature, perfusion flow, axial deformation, and frequency) with simultaneous feedback control of temperature and pH values within the cell culture.

### Tuning bioreactor system operation parameters according to chitosan-graphene mechanical properties

An *in silico* evaluation was carried out to estimate the shear and compressive stresses affecting the tissue constructs as a result of specific flow rate/axial deformation values (Table 1). Parameters resulting in a compressive stress corresponding to the lowest με physiological value (where με is the unit of the fractional change in dimension, within the range of 100–2000 με)^28^ were used as operating inputs for bioreactor system tuning.

**Table 1:** Specific shear and compressive stress values [Pa] resulting from different perfusion flow rates and compressions.

		Flow rate [mL/min]					
		0	1	2	3	4	5
Axial deformation [%]	**0**	—	5.95E-06	1.19E-05	1.79E-05	2.38E-05	2.97E-05
	**1**	2.08	2.08	2.08	2.08	2.08	2.08
	**2**	4.16	4.16	4.16	4.16	4.16	4.16
	**3**	6.23	6.23	6.23	6.23	6.23	6.23
	**4**	8.31	8.31	8.31	8.31	8.31	8.31
	**5**	10.39	10.39	10.39	10.39	10.39	10.39

Data are expressed as average values and are calculated as the sum of the stress values measured at each point of the mesh divided by the number of points.

Figure 3b shows the finite element model (FEM) geometry for the culture chamber (grey) and the scaffold (blue) used for the above assessment in our study, where CHT/G mechanical properties are assigned. Perfusion flow rate in the range of 1 to 5 mL/min, compression providing 1 to 5% axial deformation, and combinations of the above-mentioned values were tested. The measured outcomes are listed in Table 1.

Perfusion flow rates resulted in average shear stress values in the range of 10^−6^ to 10^−5^ Pa. The axial deformation range explored resulted in average higher compressive stress values in the range of 100 to 10^1^ Pa (corresponding to a strain in the range of 190 to 935 με). The addition of perfusion flow did not affect the range of the average compressive stress values. A representative display of how perfusion flow (1 mL/min), compression (1% axial deformation), or perfusion and compression together (using the above-mentioned conditions) impact the scaffold is presented in Fig. 3c–e. The colour bar shows a relative range of stress levels from lower (blue) to higher (red) values. Figure 3c shows the shear stress values occurring onto scaffolds fibres with only perfusion applied. The resulting light blue color shows low shear stress, with average values of about 10^−6^ Pa. On the other hand, Fig. 3d,e, where compression was applied either alone or in combination with perfusion, show similar yellow/orange colour distributions, which are associated with higher compressive stress values (average of about 100 Pa) than those elicited when only perfusion was applied. These results imply that adding perfusion flow appears not to affect the range of the average compressive stress values.

### Bioreactor system culture effect on cell viability and matrix mineralization

Following the *in silico* evaluation (Table 1), the lowest average shear and compressive stress simulated value (2.08 Pa), obtained with a perfusion flow of 1 mL/min and a compression of 1% axial deformation at a frequency of 1 Hz, was used to set the bioreactor operational parameters, in accordance with other previously published protocols^23–27^. After an initial 7 days under traditional static culture conditions to promote hBMSC adhesion and proliferation over/within twelve CHT/G scaffolds, six TEed constructs were exposed to continuous perfusion flow within our device with compression applied twice daily for 30 min for 7 days. The remaining six constructs were maintained for the same time under traditional static culture conditions as a reference control to evaluate the effects of the bioreactor system on cell viability and ECM mineralization. One scaffold from the bioreactor system and one maintained in static condition were evaluated by the LIVE/DEAD^®^ assay to measure cell viability.

Figure 5 shows cell distribution and viability inside the CHT/G scaffolds, comparing the static (Fig. 5a,b) and dynamic (Fig. 5c,d) cultures. Figures 5a through 5d qualitatively show the increased viability of hBMSCs under dynamic vs static conditions. Larger numbers of viable cells were obvious under dynamic culture conditions, along with consistently reduced numbers of dead cells, when compared to a traditional static culture protocol. A quantitative analysis of live or dead cells, in static (Fig. 5e,f) vs. dynamic (Fig. 5g,h) conditions within the outer (Fig. 5e,g) and inner (Fig. 5f,h) layers of the scaffold, was performed via maximum entropy threshold-based image segmentation to confirm this qualitative evidence. Figures 5i,j show the areas of the scaffolds that contain viable or dead cells. The effect of perfusion exchange enhancement under dynamic culture conditions was particularly evident within the inner scaffold core, where significantly more viable and less dead cells were present than under traditional culture conditions. On the other hand, an apparent absence of statistically significant differences between static and dynamic culture conditions was observed in the outer scaffold layers.

To evaluate the bioreactor system culture effect on early commitment of the hBMSCs towards the osteogenic phenotype, mineralization was evaluated in TEed constructs via von Kossa staining of their texture, after 7 days under dynamic or static culture.

Figure 6 shows von Kossa staining within the core of static (Fig. 6a,b) and dynamic (Fig. 6c,d) culture constructs. The qualitative optical microscopy images (Fig. 6a,c) show a larger number of von Kossa black spots under dynamic conditions. This was confirmed by performing a quantitative analysis using the maximum entropy threshold-based image segmentation method; the total amount of scaffold area covered by the von Kossa black spots in static vs. dynamic conditions are shown in Fig. 6b,d, respectively. ECM mineralization was then quantified under static and dynamic (perfusion and compression) conditions (Fig. 6e). A statistically significant (p < 0.05) effect of the compression stimulus was evident within the core of the 3D CHT/G scaffolds maintained under dynamic culture conditions, where sizable ECM mineralization is present compared to under traditional culture conditions.

## Discussion

Although TEed bone tissue has been viewed as a potential alternative to the conventional use of natural bone grafts, its effective translation to clinical practice is still impractical. Likely responsible for the delayed progress towards such a process are poorly standardized applicative protocols. In other words, there is still a need to define minimal expansion and commitment parameters to proceed towards successful mesenchymal stem cell (MSC) transfer, homing, and differentiation into the host tissue. Over the past decade, tissue engineering (TE) has undergone significant progress driven by the convergence of biology, materials science, chemistry, and engineering strategies^29^. As a consequence, current biofabrication technologies have enabled the manufacturing of 3D artificial tissue constructs. In this respect, a TE approach to heal bone defects will greatly profit from bone grafts based on substitutes primed *in vitro* and able to integrate with the host, where they will reach full final differentiation^30^.

To this aim, various biomaterials - both biomimetic synthetic polymers and biological molecules - manufactured via several fabrication techniques, have been used as ECM substitutes with adequate biological and mechanical properties able to provide support for cell attachment, proliferation, and differentiation^31^. Autologous human MSCs (more specifically, bone marrow-derived MSCs, or hBMSCs) are suitable candidates to populate scaffolds, as they show evidence of sustained osteogenic capacity^32^. Indeed, given their proliferation potential, biomolecular production, cell-to-cell signalling, and formation of the appropriate ECM, they efficiently differentiate down the osteogenic path and also secrete paracrine factors that may aid in the survival and vascularization of engineered bone^33^. Mechanostimulation acts as a significant input to maintain and/or induce the bone phenotype^34^, while increasing culture diffusion regimens^35^. In particular, compressive loads act as a key physical cue in natural bone, significantly increasing osteogenic markers^7^, and can be delivered over cells onboard of a scaffold to address their phenotype commitment. Thus, a combination of appropriate cells, biomaterials/scaffolds, and physical stimulation can be considered a successful approach to translate bone TE into clinics.

A standard protocol to induce MSCs onboard of a 3D scaffold towards osteogenic lineage commitment is crucial. Starting from this paradigm, the aim of this study was to develop a novel perfusion/compression bioreactor system as an efficient tool to standardize bone TE. Based on this idea, a bioreactor was designed as a compact unit including perfusion/compression in a standalone culture apparatus, which also features innovative properties such as automatic media replacement and removable culture units intended for real-time monitoring of developing TEed constructs. The use of Spiros^®^/MicroClave^®^ locks, to easily connect/disconnect a culture chamber to/from the perfusion line, was designed to enable the user to easily perform specific evaluations of interest (e.g. spectrofluorometric analysis, fluorescence analysis, or X-ray μCT for 3D imaging analysis) on a given developing TEed construct at different time points during culturing. Perfusion was aimed at improving diffusive exchanges, i.e. nutrient uptake and waste product removal, while compression was applied to exert mechanical cues over the cells onboard the scaffold to address their phenotype commitment. The standalone apparatus was conceived in such a way as to avoid the use of a dedicated cell culture incubator: that is, to allow the use of the device on a standard lab benchtop.

Culture parameters (i.e. a temperature of 37 °C and pH of 7.4) were initially monitored for a week in order to verify the stability of the system (i.e. without detecting any drift in the parameters). Bioreactor component sterilization was carried out prior to beginning the culturing, and no contamination was detected during test period. Besides minimizing contamination, another benefit of the automatic media replacement protocol is that it allows the progressive replacement of the media (e.g. at a rate of 1 mL/hr), avoiding the stress on the cell culture that results from the bulk media changes typically done within traditional subculture procedures^36^.

An *in silico* evaluation was carried out to estimate the shear and compressive stresses affecting the scaffolds, and therefore the cells onboard, when perfusion and/or compression are applied. Ranges for perfusion flow (1 to 5 mL/min) and compression (1 to 5% axial deformation) rates were selected based on published evidence^23–27^. No apparent turbulence was detected under these conditions. The resulting average shear and compressive stress values were estimated to be in the range of 10^−6^ to 10^1^ Pa, depending on the perfusion and compression conditions applied. The average compressive stress value, obtained under application of 1% axial deformation, was approximately six orders of magnitude higher than the average shear stress induced by applying only perfusion stimulus at 1 mL/min. This implies that when both perfusion and compression are applied, the perfusion shear stress is negligible compared to the compressive stress. Accordingly, a perfusion flow rate of 1 mL/min was applied during the experimental dynamic culture with the purpose of maintaining appropriate diffusive exchanges^8^. In addition, dynamic compression was applied twice per day, for 30 min each time, with a 1% axial deformation at 1 Hz; this resulted in a strain of 190 με, which is at the lower end of the useful range to address osteogenic phenotype commitment, reported in the literature to be 100 to 2000 με^28^. In particular, a small number of loading cycles (i.e. 24 to 100 cycles per day), with frequencies in the range of 0.5 to 1 Hz, resulted in up to a 24% increase in bone cross-sectional area and up to a 45% increase in bone mineral content. The effects of perfusion and compression stimuli on cell viability and ECM mineralization were evaluated at different time points. Proliferation was investigated on day 3 of the dynamic culture period, i.e. day 10 of the entire culture protocol, through a cell viability assay. Both qualitative and quantitative data showed a larger amount of viable cells under dynamic culture conditions, together with a consistently reduced number of dead cells when compared to a traditional static culture protocol; it is hypothesized that this is the result of the contribution of perfusion to improving nutrient exchange and waste removal. The improved cell viability suggests that cell growth and proliferation are more adequately sustained in our system than under standard culture conditions, in particular when nutrient diffusion into the core of a 3D TEed construct was considered.

Perfusion flow-induced shear stress is unlikely to be responsible for dead cell detachment, given its negligible values (Table 1) and the scaffold’s “shield” effect over the cells. The level of early ECM mineralization was evaluated through von Kossa staining; qualitative and quantitative data confirmed that our bioreactor system transferred a suitable mechanical cue to the scaffold, which was translated into a sizable biological effect. Such an evidence asks for a future application of our bioreactor system to test different biomaterial scaffolds and cell lineages. We plan this fulfillment using a second generation device overcoming some limitations, i.e. its encumbrance and the Plexiglas wear, experienced with the present prototype.

## Conclusions

We developed and tested a novel standalone bioreactor system suitable to apply different perfusion (1 to 5 mL/min) and compression (1 to 5% axial deformation at frequencies of 0.5 to 5 Hz) stimuli, to transfer significant inputs to hBMSCs seeded onto a 3D scaffold. Perfusion improved nutrient exchange and waste removal, resulting in increased cell viability, while cyclic compression loading enhanced ECM mineralization. These observations show that our novel device could be a potentially valuable a technology for addressing and investigating the osteogenic phenotype commitment of stem cells, both in view of possible clinical applications^37^ and for basic laboratory research protocols.

## Materials and Methods

### Bioreactor system design

#### Finite element modelling of stress distribution in a dynamic culture

A finite element model (FEM, COMSOL Multiphysics® Modeling Software, Stockholm, Sweden) was implemented to assess the stress distribution into 3D cell/scaffold constructs under perfusion and/or compression stimuli. All the components were obtained using primitive geometries and Boolean operations. The inner chamber had a diameter of 6 mm and a height of 9 mm. The scaffold was 5 mm in diameter and 6 mm in height. The inlet/outlet diameter was 3.2 mm. To approximate the medium properties, the inner volume of the well was modelled as water. Navier-Stokes equations were set for the viscous fluid. Darcy’s law was used for the laminar fluid flow. Linear elasticity equations were set for the scaffold. A sensitivity study of the mesh was performed in order to obtain the most computationally efficient solution. The specific parameters listed in Table 2 were measured experimentally.

**Table 2:** FEM parameters for 3D cell/scaffold constructs.

Parameter	Value	Units
Permeability	7E-11	m^2^
Porosity	0.71	—
Young’s modulus	2E Em04	Pa
Poisson ratio	0.8	—
Density	60	kg/m^3^

Perfusion flow rates in the range of 1 to 5 mL/min, compression within 1 to 5% axial deformation, and combinations of above-mentioned values were tested in order to estimate the operating inputs for bioreactor system tuning.

### Bioreactor system architecture

A perfusion/compression bioreactor was designed and built with the aim of improving the growth and the osteogenic commitment of the stem cells seeded over a scaffold of interest. The bioreactor is a standalone system that can automatically run perfusion and compression protocols while monitoring and maintaining a stable temperature and pH. The Control Unit (CU) of the system is based on a micro-controller (ATmega2560, Atmel Corp., USA), which communicates with a Graphical User Interface (GUI) (implemented in LabView, National Instruments, USA) to configure the parameters of the system.

#### Culture chamber layout

Custom chambers, which host tissue constructs and allow perfusion and compression over cell-seeded 3D scaffolds, were fabricated to follow the geometry of a standard 96-plate single well (i.e. a cylinder with 300 µL volume) and 3D printed in a polymeric biocompatible material (TuskT^®^, Materialise HQ, Leuven, Belgium). Each chamber represents an independent detachable unit integrated into the perfusion line via Spiros/MicroClave connectors (ICUMed, San Clemente, CA, USA), as illustrated in Fig. 3a.

#### Standalone culture apparatus set-up

Two WPX1 peristaltic pumps (WELCO Co. Ltd., Tokyo, Japan) were used to supply fresh medium to and remove exhausted medium from the bioreactor circuit. The system controls both pumps to provide automatic handling of the fluid exchange, which facilitates medium replacement while decreasing culture contamination risk due to human intervention.

The system temperature was maintained at 37 °C by a closed-loop control utilizing three heating pads distributed in key segments of the circuit and the feedback from a DS18B20 thermistor (Dallas Semiconductor, Dallas, TX, USA). Using a similar strategy, a gas mixture of 5% CO_2_, 20% O_2_, and 75% N was bubbled into the medium to guarantee a pH level of 7.4 pH, which was monitored by a BNC pH lab electrode probe (Phidgets Inc., Calgary, AB, Canada).

#### Perfusion/compression actuation design

A WP10 peristaltic pump (WELCO Co. Ltd.) was implemented to provide continuous nutrient delivery and waste removal to/from the TEed constructs. The perfusion flow rate can be adjusted up to 5 mL/min per single culture chamber, in accordance with referenced values^25^.

A NEMA-17 hybrid bipolar stepping motor (Songyang Machinery & Electronics Co. Ltd., Changzhou, China) enables controlled cyclic compression of the TEed scaffolds. The system allows the compression to be configured within the range of 1 to 5% at frequencies of 0.5 to 5 Hz. The parameters are set via the GUI and controlled automatically by the CU.

### 3D cell culture setting and analysis

#### Human mesenchymal stem cell culture

Primary human bone marrow-derived mesenchymal stem cells (hBMSCs) were purchased (Lonza Inc., Allendale, NJ, USA) and used at passage #2 for the seeding onboard of 3D scaffolds and the subsequent 1 week culture either within our bioreactor system or under conventional static conditions as a control. To this aim, the cells were initially expanded as a 2D monolayer in high glucose Dulbecco’s Modified Eagle Medium (DMEM) containing 0.1% penicillin/streptomycin, 0.1 mM non-essential amino acids, and 10% foetal bovine serum. After two passages, the hBMSCs were trypsinized and counted in a haemocytometer using Trypan Blue staining to evaluate the number of dead cells. All reagents were purchased from Life Technologies (Carlsbad, CA, USA).

#### 3D chitosan-graphene scaffolds

Graphene oxide was prepared according to Hummers and Offeman^38^ at the National Institute for Research and Development in Microtechnologies (Romania). Acetic acid (99.7%) and chitosan (CHT) from crab shells were purchased from Sigma-Aldrich (St Louis, MO, USA). All materials were dissolved without further purification in double distilled (dd) water. 2.5 g CHT was mixed with 250 mL acetic acid solution (10% v/v in dd water) at 50 °C in order to form a homogeneous viscous solution. Further, graphene oxide, at a concentration of either 0.5 or 3 g/100 mL (w/v), was added to the CHT solution and mixed by ultrasonication for 1 h at room temperature (RT). The resulting homogeneous solution was casted onto transparent glass Petri dishes, then frozen overnight at −70 °C and freeze-dried for 2 days at −50 °C and 0.040 mbar. After sublimation of ice crystals by freeze-drying, the polymer structure became porous. The 3D dried materials were thermally treated under vacuum, according to the following procedure: 50 °C for 30 min, 70 °C for 30 min, and 90 °C overnight. The obtained samples were then subjected to an advanced characterization of the bulk surface and to an *in vitro* biocompatibility assessment according to Meloan and Puchtler^39^.

#### 3D cell culture

Chitosan-graphene (CHT/G) scaffolds (200 mL volume each) were seeded with hBMSCs (1 × 10^6^ cells/mL) in a standard 24-well plate and maintained at conventional static culture conditions for 1 week. Afterwards, the scaffolds were placed either into the bioreactor system culture chambers or maintained under conventional static culture conditions, as above. The hBMSCs were then cultured for 1 additional week, in either dynamic or static culture, for a total of 14 days of culturing. The medium was formulated in high glucose DMEM, with 40 mg/mL proline (Sigma-Aldrich), 10% human platelet lysate (as a substitute for the traditionally-used foetal bovine serum as a soluble growth factor source; platelet lysate was obtained from the Blood Bank of the Landspitali University Hospital, Reykjavik, Iceland), and 0.1% penicillin/streptomycin (Life Technologies). The medium was supplemented with an osteogenic culture mix to provide final concentrations of 0.1% sodium pyruvate (Life Technologies), 50 mg/mL ascorbate 2-phosphate (Sigma-Aldrich), 0.1 mM dexamethasone, 1% ITS M premix (BD Biosciences, Bedford, MA), and 10 ng/mL TGFβ-3 (R&D Systems, Minneapolis, MN). The medium was changed every 3 days in both culture protocols. Perfusion/compression regimens applied to 3D cell/scaffold constructs in dynamic culture were identified through a FEM and are detailed further below.

#### LIVE/DEAD® cell viability assay

100 μL of 2 μM calcein AM / 4 μM EthD-1 (Thermo Fisher Scientific, Waltham, MA, USA) in sterile tissue culture–grade phosphate-buffered saline (PBS) was administered on top of a microtome (cross section) of cellularized CHT/G scaffold, cut with a scalpel. Three sections, placed on top of a microscope coverslip, were incubated within a covered Petri dish, to prevent drying of the samples, for 1 h at RT in darkness. Following incubation, the samples were washed with PBS. Each wet coverslip was then mounted on top of a microscope slide and sealed with fingernail polish. Each sample was analysed under a fluorescence microscope. Green calcein fluorescence (Ex/Em 494/517) was emitted by living cells, whereas Red EthD-1 fluorescence (Ex/Em 528/617) indicated dead cell nuclei.

#### von Kossa staining

A standard von Kossa staining protocol^40^ was used to quantify the deposition of calcium/calcium salts occurring during ECM mineralization. Microtome sections from cellularized CHT/G scaffolds were fixed on the top of microscope slides in cold methanol for 15 to 20 min. After three PBS rinses, the slides were incubated with 5% silver nitrate solution under UV light. Reduced calcium could then be identified via the presence of metallic silver nodules, which appeared as black spots.

#### LIVE/DEAD® and von Kossa quantitative analysis

Quantitative analysis of cell viability and ECM mineralization was performed on images acquired from cellularized CHT/G scaffold slices. Control (static culture) and dynamic (bioreactor system) data were compared. To this aim, images acquired with a Nikon TE 2000U optical microscope were processed, using ImageJ software (National Institute of Health, USA) in order to determine the size of the green or red (LIVE/DEAD^®^ assay) and black (von Kossa staining) areas. These areas were calculated using the maximum entropy threshold-based image segmentation method. The total area (A) covered by specific spots of interest in the total scaffold slice surface was calculated using the Eq. (1),

1 $${A}={np}\cdot {p}{{d}}^{2}$$

### Statistical analysis

A Shapiro-Wilk normality test was performed to confirm that the data followed a normal distribution. Student’s t-tests were used to determine significant differences between groups. The significance level was set at p <0.05. Results are presented as mean ± standard deviation.
